# Laparoscopic resection of an intra-abdominal esophageal duplication cyst in the ileum: a case report

**DOI:** 10.1186/s40792-022-01576-6

**Published:** 2022-12-09

**Authors:** Shosaburo Oyama, Kenji Tanaka, Masaaki Moriyama, Takashi Nonaka, Tetsuro Tominaga, Terumitsu Sawai, Naoe Kinoshita, Takeshi Nagayasu

**Affiliations:** 1 Department of Surgery, Saiseikai Nagasaki Hospital, 2-5-1 Katafuchi, Nagasaki, 850-0003 Japan; 2 Department of Surgical Oncology, Nagasaki University Graduate School of Biomedical Sciences, 1-7-1 Sakamoto, Nagasaki, 852-8501 Japan; 3 Department of Diagnostic Pathology, Saiseikai Nagasaki Hospital, 2-5-1 Katafuchi, Nagasaki, 850-0003 Japan

**Keywords:** Intra-abdominal esophageal duplication cyst, Ileum, Laparoscopic surgery

## Abstract

**Background:**

Esophageal duplication cyst (EDC) is a type of gastrointestinal duplication cyst that involves congenital malformations of the gastrointestinal tract. EDCs are frequently found in the mediastinum and thoracoabdominal region, but rarely occur in the abdominal cavity. However, intra-abdominal EDCs are frequently found in the upper abdomen near the abdominal esophagus. Here, we report, for the first time, a case of intra-abdominal EDC that occurred in the ileum.

**Case presentation:**

A 14-year-old female patient presented to our hospital with complaints of epigastric pain and vomiting. Abdominal computed tomography (CT) revealed a cystic tumor in the pelvis, suspected of ovarian origin. She was admitted to our gynecology department and underwent emergency surgery. The laparoscopic examination revealed that both ovaries were intact and that a primary tumor had developed from the ileal mesentery. Since the patient’s condition was not urgent at the time of the gynecological surgery, the procedure was completed by only performing exploratory laparotomy; the patient was admitted to our department after the surgery. Pelvic magnetic resonance imaging performed on the next day revealed a cystic mass measuring 90 × 65 mm with a smooth margin and homogeneous signal intensity, arising posterior to the uterus. The mass was suspected as an intestinal duplication cyst. On another day, after the examinations were completed, we resected the portion of the small intestine containing the tumor by laparoscopy. The patient had a successful postoperative course and was discharged on the 5th postoperative day. Histological examination showed that the cyst was lined by stratified squamous epithelium, contained esophageal glands, and had a two-layer muscularis propria. Therefore, a diagnosis of intra-abdominal EDC was performed.

**Conclusions:**

An intra-abdominal EDC cyst is relatively rare; this is the first case reported at the distal ileum.

## Background

Esophageal duplication cysts (EDCs) are alimentary duplications and rare congenital malformations [1]. Gastrointestinal duplication is an abnormal development of the gastrointestinal tract involving the formation of spherical or tubular structures either in continuity or adjacent to the gastrointestinal tract [2]. Gastrointestinal duplication frequently occurs in the small and large intestines; however, EDCs occur most commonly near the esophagus. Most reports have described EDCs developing from the cervical to the abdominal esophagus. EDCs rarely occur outside the esophagus and are particularly rare in the upper abdomen (around the pancreas and duodenum) [3, 4].

We encountered a case of an intra-abdominal EDC that occurred at the distal ileum. This is a rare case because the esophageal tissue (which is of foregut origin) was found in the terminal ileum (which is of midgut origin). Embryologically, the esophageal tissue is hypothesized to become an ectopic lesion after invading the ileum during development, as in the ectopic pancreas [5]. In addition, the findings, in this case, are consistent with the histopathological findings, which support the diagnosis of an EDC. Based on the summary of previous literature, this is the first reported case of an EDC occurring at the distal ileum.

## Case presentation

**Fig. 1:**
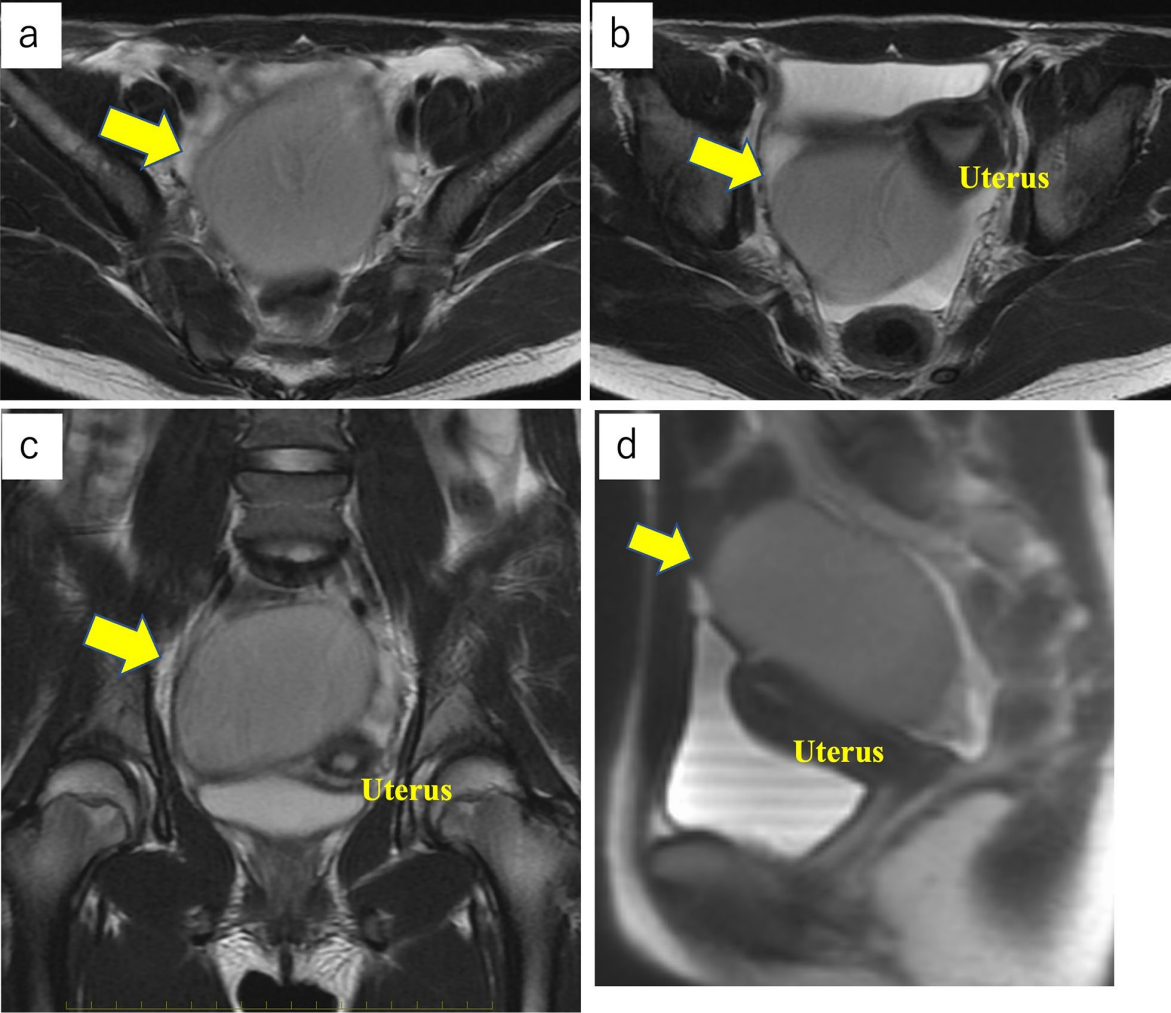
Preoperative pelvic magnetic resonance imaging. Preoperative images show a tumor (yellow arrow) that was a cystic mass measuring 90 × 65 mm with a smooth margin and homogeneous signal intensity on the right dorsal side of the uterus (**a**–**d**). Coronal image (**c**), sagittal image (**d**). T1-weighted images present slightly higher signals than those of water, and T2-weighted images show low signals. No apparent communication was found between the cyst and the intestinal tract

**Fig. 2:**
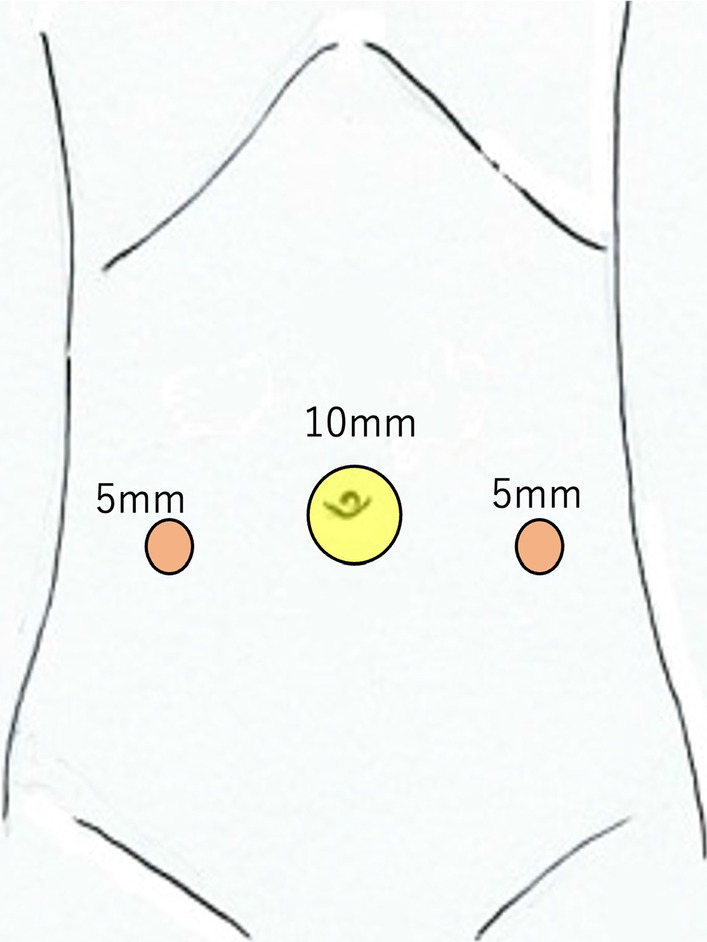
Port site placement. The image shows the three ports of laparoscopic surgery. The umbilical port is a 12-mm camera port (yellow circle), and the bilateral abdomen port is a 5-mm working port for handling the forceps (red circle)

**Fig. 3:**
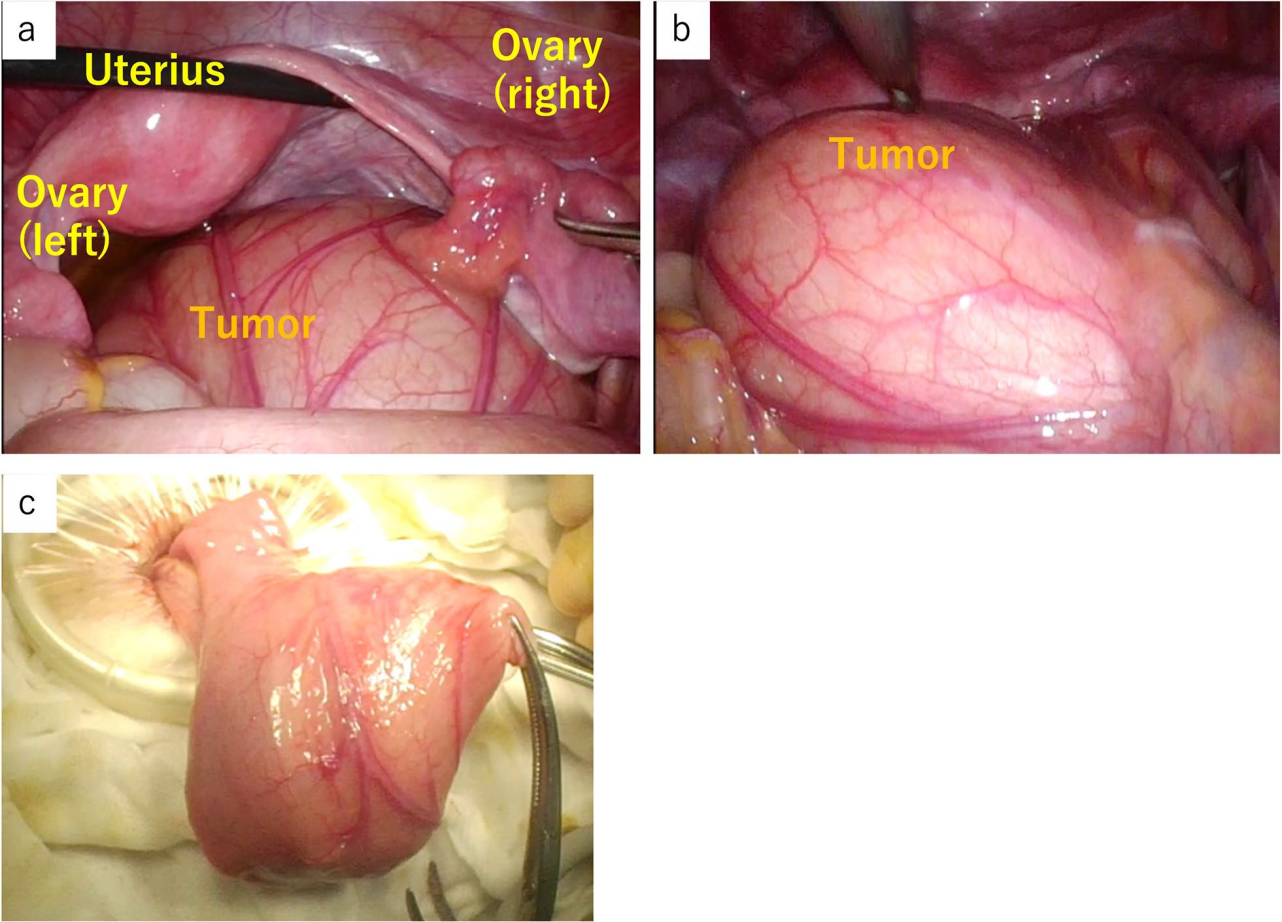
Intraoperative laparoscopic view. The image shows a tumor as a lesion that fits into the pelvis (**a**). The lesion has good mobility without adhesions in the pelvis and can exit the body cavity from the umbilical region (**b**, **c**). The cystic tumor was located on the mesenteric side, approximately 40 cm proximal to the distal ileum. The boundary with the adjacent intestinal tract is unclear in the intraoperative findings, and partial resection of the small intestine containing the tumor was performed

**Fig. 4:**
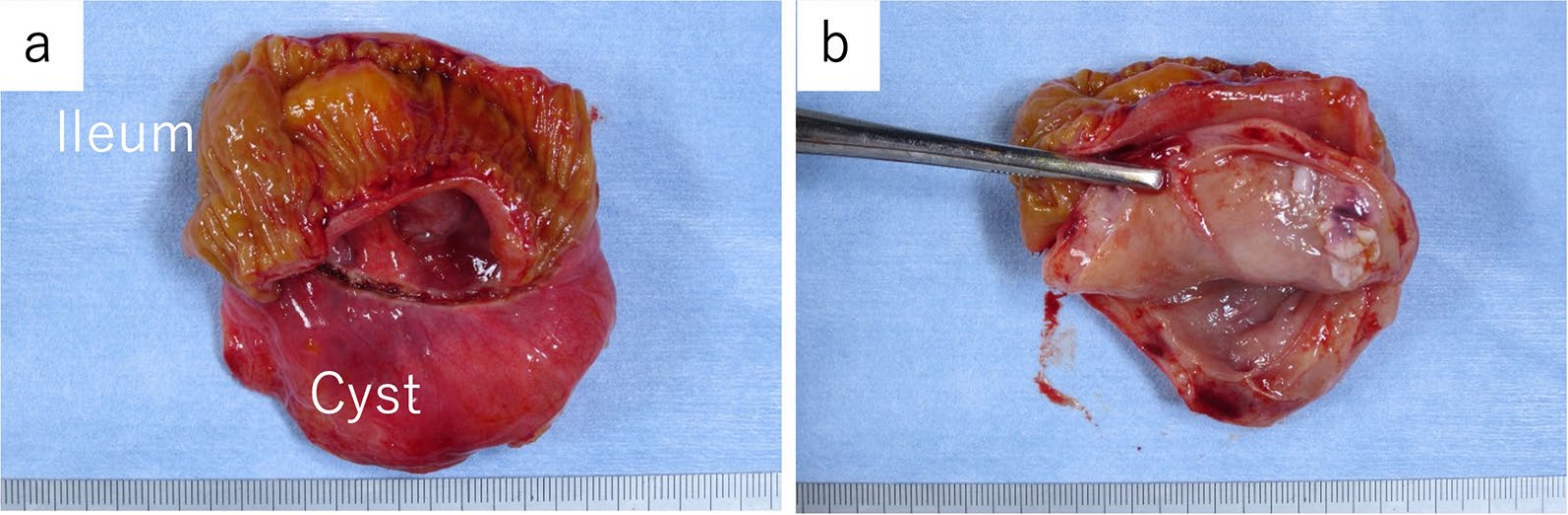
Extracted specimen. The image shows the specimen at the ileal lumen (**a**). No change in the lumen and no apparent communication were observed with the cyst. The image shows the lumen of the cyst (**b**)

A 14-year-old female patient with no significant medical history presented to our hospital with complaints of epigastric pain and vomiting for a few days. Computed tomography (CT) scan showed a tumor in the pelvis. The right ovary was visible rather than in the left ovary. She was admitted to our gynecology department due to a suspected ovarian tumor and underwent emergency surgery. A laparoscopic examination of the pelvic cavity revealed normal ovaries bilaterally and a tumor originating from the ileal mesentery. Since the patient’s condition was not urgent at the time of the gynecological surgery, the procedure was completed by only performing exploratory laparotomy. Subsequently, the patient was referred to our department for a detailed examination. Contrast-enhanced pelvic CT was performed on the next day, which revealed a 90 × 65-mm cystic mass with a smooth margin and internal homogenous density, arising posterior to the uterus. Contrast-enhanced pelvic magnetic resonance imaging (MRI) revealed a mass that showed slightly higher signals than those of water on T1-weighted images and lower signals on T2-weighted images (Fig. 1). In addition, the MRI findings showed no evidence of communication between the cyst and the adjacent intestinal tract. Laparoscopic surgery was performed for a tentative preoperative diagnosis of intestinal duplication. Subsequently, we performed a three-port laparoscopic surgery (Fig. 2). The umbilical port was a 12-mm camera port, and the other two ports were used for handling the forceps. Intraoperative findings revealed a primary cystic tumor of the ileal mesentery in the pelvis, which was soft, elastic, and mobile with no adhesions. Subsequently, the umbilical port wound was extended to a 4-cm small laparotomy incision, and a wound protector (S) was attached. Finally, partial intestinal resection was performed by removing the portion of the small intestine containing the tumor and pulling it out of the abdominal cavity through the small umbilical laparotomy wound (Fig. 3). The excised specimen showed no communication between the cyst and intestinal tract (Fig. 4). Postoperatively, patient symptoms resolved, and her condition was stable; therefore, she was discharged on the 5th postoperative day.

**Fig. 5:**
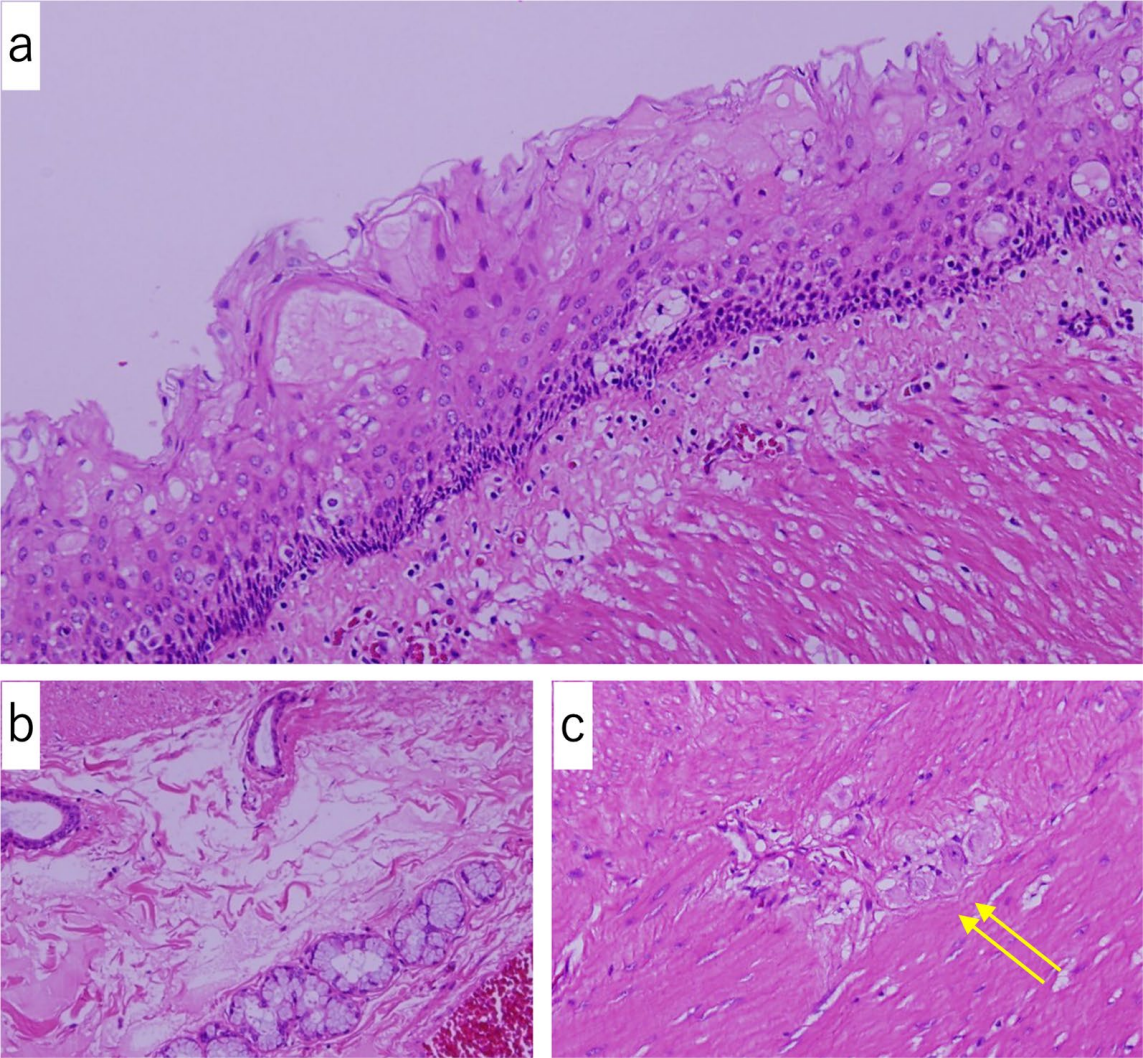
Histopathological findings. The cystic epithelium appears as stratified squamous epithelium (hematoxylin–eosin [HE], ×100) (**a**). The esophageal glands are observed in the submucosa, which has mucous glands and ducts (HE, ×100) (**b**). The muscular layer has a two-layer structure with inner circular and outer longitudinal muscle layers (**c**). In addition, nerves and ganglion cells (Auerbach plexus, yellow arrow) are found between the two layers (HE, ×100)

Histological examination of the specimen showed that the cyst wall was lined by stratified squamous epithelium (Fig. 5a) and shared the smooth muscle of the intact intestine. Several esophageal glands were also observed (Fig. 5b). In addition, the muscular layer had a two-layer structure—the inner circular and outer longitudinal muscle layers—with nerves and ganglion cells (Auerbach plexus) between the layers. There was no evidence of cytologic epithelial atypia or malignancy, and no cartilage or bone tissue was found. Therefore, the condition was diagnosed as intra-abdominal EDC. The patient was followed up for 5 years postoperatively and was recurrence-free.

## Discussion

EDC is an abnormal esophageal development that occurs during the 5th to the 8th gestational weeks when the primitive foregut forms the gastrointestinal tract [4, 6–8]. A previous report showed that EDCs are frequently found in the mediastinum and thoracoabdominal region but rarely in the abdominal cavity and that intra-abdominal EDCs are frequently observed in the upper abdomen near the abdominal esophagus [9]. In contrast, the esophageal tissue was found in the ileum in this case. Various theories exist on the mechanism underlying EDC development [7, 10]. In this case, it is more likely that the esophageal tissue may become an ectopic lesion after invading the ileum during development. A similar condition is an ectopic pancreas, where the pancreatic tissue (which is of foregut origin) is ectopically found in the small intestine (which is of midgut origin) [5].

The pathological criteria for EDCs are as follows [11]: (1) attachment to the esophageal wall, (2) presence of stratified squamous epithelium, and (3) presence of two layers of muscularis propria. According to several reports, the first criterion is not always necessary to diagnose EDCs [3, 4, 9, 12]. However, this case met the second and third criteria. Furthermore, the absence of cartilage and bone tissue led to the diagnosis of EDC. This sporadic case proves that intra-abdominal EDC can occur in the ileum.

There are only 25 reported cases of intra-abdominal EDCs [1–4, 6–10, 12–24], including our case. We have summarized these reports in Table 1. In recent cases, intra-abdominal EDCs occurred in the upper abdomen, except in this case, with a median age of 50 years (range, 1 month to 70 years). This lesion has been reported in 9 men and 16 women. Most cases of intra-abdominal EDCs are asymptomatic; however, symptoms such as epigastric pain and nausea are occasionally present. The median maximum diameter of the tumor is 45 mm. Evidence suggests that as the tumor grows, it causes gastrointestinal obstruction, bleeding, perforation, and abscess formation. The tumor is frequently found on abdominal imaging when examining for abdominal symptoms, and a preoperative diagnosis is rarely performed. Moreover, it is frequently found on postoperative histological examination. Due to recent developments in imaging examinations, abdominal cystic masses can be detected before birth in some cases [24].

**Table 1:** Characteristics of 25 published cases of intra-abdominal esophageal duplication cyst (EDC), including our case

Reference	Age	Sex	Symptom	Location	Maximum tumor size	Treatment	Postoperative hospital day	Recurrence
Ruffin et al	38 years	F	Epigastric pain, nausea, and vomiting	Distal esophagus	40 mm	Laparotomy	N/A	N/A
Harvell et al	57 years	F	Epigastric pain	Pancreas	22 mm	Laparoscopic resection	N/A	N/A
Karahasanoglu et al	51 years	M	Dysphagia, weight loss, and epigastric pain	Distal esophagus	110 mm	Laparotomy	10	No recurrence
Janssen and Fiedler	56 years	F	Decreased appetite, weight loss	Superior to the left kidney	80 mm	Biopsy	N/A	N/A
Rathaus et al	5 years	F	Epigastric pain	Distal esophagus	10 mm	Laparotomy	N/A	N/A
Nelms et al	44 years	M	Low back pain	Distal esophagus	70 mm	Laparoscopic resection	N/A	N/A
Vijayaraghavan et al	70 years	F	Retching, giddiness, headache	Distal esophagus	75 mm	Laparotomy	N/A	N/A
Noguchi et al	26 years	F	N/A	Distal esophagus	40 mm	Laparoscopic resection	8	No recurrence
Kin et al	51 years	F	N/A	Distal esophagus	45 mm	Laparoscopic resection	N/A	N/A
Kim et al	52 years	F	N/A	Distal esophagus	40 mm	Laparotomy	9	No recurrence
Martin et al	60 years	M	Epigastric pain, gastric outlet obstruction	Duodenum	100 mm	Laparotomy	3	No recurrence
Martin et al	50 years	F	Left side flank pain	Pancreas	65 mm	Laparotomy	N/A	No recurrence
Naritaka et al	57 years	M	Epigastric pain	Distal esophagus	50 mm	Laparotomy	16	No recurrence
Aldrink et al	2 years	M	N/A	Distal esophagus	30 mm	Laparoscopic resection	3	No recurrence
Gümüs et al	18 years	F	Dyspeptic complaints	Distal esophagus	42 mm	Laparotomy	N/A	N/A
Bhamidipati et al	69 years	M	N/A	Distal esophagus	44 mm	Laparoscopic resection	3	No recurrence
Pujar et al	13 years	F	Epigastric pain and vomiting	Distal esophagus	50 mm	Laparoscopic resection	6	N/A
Mori et al	9 years	M	N/A	Distal esophagus	22 mm	Laparoscopic resection	8	No recurrence
Castelijns et al	20 years	M	Nausea and colic pain	Distal esophagus	30 mm	Laparoscopic resection	2	No recurrence
Huang et al	20 years	F	N/A	Stomach	138 mm	Laparoscopic resection	8	No recurrence
Watanabe et al	50 years	M	Epigastric pain and dysphagia	Distal esophagus	35 mm	Laparoscopic resection	10	No recurrence
Khatib et al	1 week	F	N/A	Distal esophagus	N/A	Laparotomy	N/A	No recurrence
Khatib et al	1 week	F	N/A	Distal esophagus	N/A	No surgery	N/A	N/A
Mori et al	64 years	F	N/A	Distal esophagus	70 mm	Laparoscopic resection	9	No recurrence
Ours	14 years	F	Epigastric pain	Ileum	90 mm	Laparoscopic resection	5	No recurrence

*N/A* not applicable

Complete resection of the tumor is the recommended treatment since EDCs may become cancerous [25, 26]. However, depending on the patient’s condition, some cases only require a puncture or biopsy and conservative treatment [12, 24, 27]. In addition, reports of recurrence exist due to the remnants of excision [28]. In our patient, the torsion of an ovarian tumor was suspected; therefore, emergency surgery was performed. Compared to the previously reported epigastric lesions, cystic lesions in the pelvis must be differentiated from gynecological diseases, and emergency surgery must be considered.

## Conclusion

This is the first reported case of intra-abdominal EDC occurring in the ileum.

## Data Availability

The datasets used or analyzed in this study are available from the corresponding author upon reasonable request.

## Abbreviations

CT: Computed tomography
EDCs: Esophageal duplication cysts
MRI: Magnetic resonance imaging
N/A: Not applicable
